# Evaluation of surface EMG-based recognition algorithms for decoding hand movements

**DOI:** 10.1007/s11517-019-02073-z

**Published:** 2019-11-21

**Authors:** Sara Abbaspour, Maria Lindén, Hamid Gholamhosseini, Autumn Naber, Max Ortiz-Catalan

**Affiliations:** 1 School of Innovation, Design and Engineering, Mälardalen University, 721 23 Västerås, Sweden; 2 RISE Acreo AB, Isafjordsgatan 22, 164 40 Kista, Sweden; 3 Department of Electrical and Electronic Engineering, Auckland University of Technology, Private Bag 92006, Auckland, 1142 New Zealand; 4 Biomechatronics and Neurorehabilitation Laboratory, Department of Electrical Engineering, Chalmers University of Technology, Gothenburg, Sweden; 5 Integrum AB, Mölndal, Sweden

**Keywords:** Electromyography, Feature extraction, Myoelectric pattern recognition, Dimensionality reduction, Classification

## Abstract

Myoelectric pattern recognition (MPR) to decode limb movements is an important advancement regarding the control of powered prostheses. However, this technology is not yet in wide clinical use. Improvements in MPR could potentially increase the functionality of powered prostheses. To this purpose, offline accuracy and processing time were measured over 44 features using six classifiers with the aim of determining new configurations of features and classifiers to improve the accuracy and response time of prosthetics control. An efficient feature set (FS: waveform length, correlation coefficient, Hjorth Parameters) was found to improve the motion recognition accuracy. Using the proposed FS significantly increased the performance of linear discriminant analysis, K-nearest neighbor, maximum likelihood estimation (MLE), and support vector machine by 5.5%, 5.7%, 6.3%, and 6.2%, respectively, when compared with the Hudgins’ set. Using the FS with MLE provided the largest improvement in offline accuracy over the Hudgins feature set, with minimal effect on the processing time. Among the 44 features tested, logarithmic root mean square and normalized logarithmic energy yielded the highest recognition rates (above 95%). We anticipate that this work will contribute to the development of more accurate surface EMG-based motor decoding systems for the control prosthetic hands.

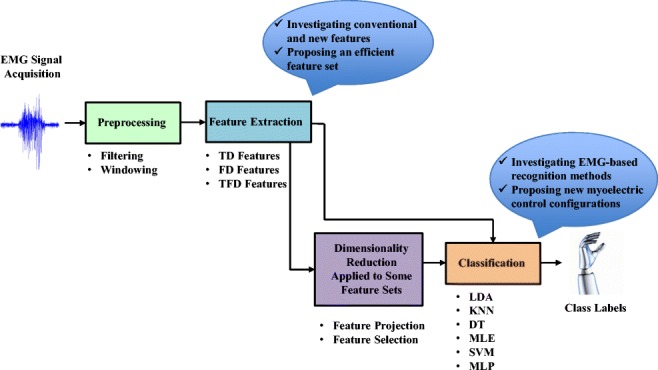

## Introduction

Myoelectric pattern recognition (MPR) controlled prosthesis ideally mimics the functionality of a natural limb using signals from residual muscles left over after amputation or congenital defect. The ability of MPR systems to decode motor volition is dependent on how well each stage of the system performs. MPR processing stages are typically divided into pre-processing, feature extraction, and classification [1]. Pre-processing is used to remove unwanted signal components from the raw electromyogram (EMG) like motion artifacts or power line interference. EMG signals are then windowed and signal features are calculated over each window. Signal features can be grouped into three categories, time domain (TD) [1], frequency domain (FD), and time-frequency domain (TFD) [2], each describing different components of the signal. If the resulting feature space is sufficiently high, dimensionality reduction techniques like principal component analysis (PCA) may be employed to improve classification accuracy and efficiency [3]. The resulting feature vectors are then fed into a classifier for training and decoding motor volition.

Various features and classification methods have been developed to improve the usability of prosthetic hands [4, 5]. Phinyomark et al. evaluated 37 of the most commonly used EMG features to discriminate hand movements using linear discriminant analysis (LDA) [6]. Based on their results, they suggested using a feature set composed of mean absolute value, waveform length, Wilson amplitude, autoregressive coefficients, and mean absolute value slope. The obtained accuracy is 92.1%. Another study by the same researchers [7] compared the performance of 50 features using LDA to classify ten upper-limb movements; they found the sample entropy feature to yield the highest classification accuracy. They also proposed a feature set of four single features to improve the accuracy up to 95%. Oskoei et al. [8] combined 12 features and used an artificial neural network (ANN) to discriminate six movements. Their study on six healthy subjects indicated that satisfactory pattern recognition accuracy could not be achieved using TD features alone. Conversely, Scheme et al. [9] used a set of TD features introduced by Hudgins et al. [10] to classify 11 classes of motion using ten commonly used classification techniques. The LDA-based, one-versus-one configuration significantly outperformed the other classifiers, achieving an error of less than 5% using TD features alone. Hargrove et al. [11] compared the classification accuracy of four different feature sets—the Hudgins TD feature set, autoregressive (AR) model, combined TD and AR (TDAR), and root mean square (RMS) using ANN and LDA classifiers—to discriminate ten classes of isometric contractions. Their results from 12 healthy subjects showed the TDAR/LDA combination had the best performance with accuracy up to 97%. Guo et al. [5] compared the classification accuracy of combinations of four TD features and ANN and support vector machine (SVM) classifiers to discriminate nine movements. They recommended muscular model (MM) and ANN for real-time applications, while MM with SVM was more suitable when processing time is not a key requirement. Although these offline studies have shown that accurate decoding of gestures from electrodes placed on the forearm can be achieved, optimal feature extraction and robust classification continue to be open challenges which likely affects the adoption and use rates of such systems by amputees for controlling prosthetic hands [12]; therefore, improvements in these areas could potentially increase the functionality and use of powered prostheses. Furthermore, due to variations in methodologies used to evaluate the algorithms in different studies, it is difficult to compare their results. Few studies have quantitatively evaluated the performance of a wide range of classifiers and features to discriminate hand and finger movements using the same database and methodology [5–8, 11].

Accuracy in decoding different motions and low response time are crucial for a successful surface EMG-based control system [13]. Therefore, the aim of the present study was to determine new configurations that improve the accuracy and responsiveness of hand gesture recognition with surface EMG signals. We investigated various combinations of 44 common and new features and six classifiers, based on the literature mentioned above, for motion recognition with surface EMG signals. We proposed a new feature set and determined new configurations for decoding individual hand movements. We examined classification accuracy and processing time using a database recorded from 20 healthy volunteers to avoid much of variability in the recordings and maintain consistency between experiments so that it is possible to compare the results of different algorithms. We anticipate that this work will contribute to the development of more accurate surface EMG-based motor decoding systems for the control prosthetic hands. The paper is organized as follows: Section 2 presents data collection and pre-processing followed by feature extractions, dimensionality reduction, and classifiers used in this study. Section 3 presents the results. Section 4 summarizes the discussion and finally, Section 5 draws conclusions.

## Methods

### Data collection

**Fig. 1:**
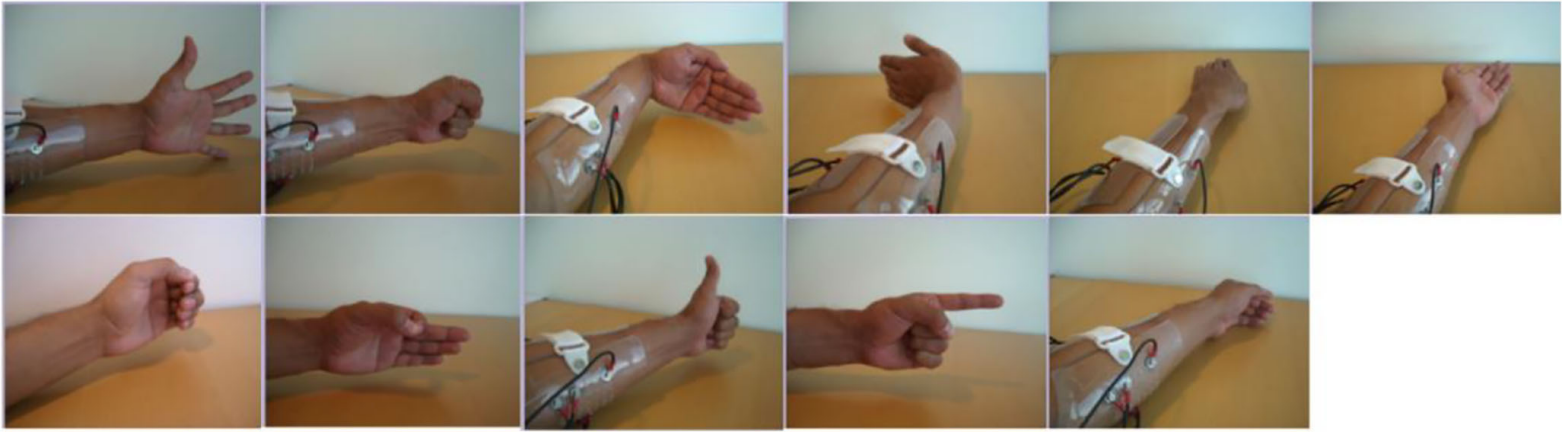
Ten classes of motions plus the relaxation or “no movement”: from top left: open hand, close hand, flex hand, extend hand, pronation, supination, side grip, fine grip, agree or thumb up, pointer or index extension, and relaxation [14]

Four channels of surface EMG signals (sampled at 2000 Hz) from 20 healthy subjects were obtained from the BioPatRec database [14]. Subjects were aged between 23 and 63 (mean ± STD 30.1 ± 10.5) years, 10 were females and 10 were males, one was left-handed and 19 were right-handed, with a mean weight of 68.8 ± 11.0 kg and a mean height of 1.77 ± 0.08 m. Recordings were taken using silver-silver chloride electrodes placed with roughly equal spacing over the proximal third of the subjects’ dominant forearm. The available hand and wrist movements in this database, as shown in Fig. 1, were open hand, close hand, flex hand, extend hand, pronation, supination, side grip, fine grip, agree or thumb up, pointer or index extension, and relaxation.

### Pre-processing

The raw EMG signals were filtered using a Butterworth band-pass filter with a bandwidth of 10–500 Hz [3, 15] and a Notch filter at 50 Hz. Afterwards, 30% of the contraction time in the EMG signal was trimmed to exclude inactive periods at the beginning and ending of the contraction (15% each) [14]. The relaxation part was added as an additional movement. Overlapping windows of 200 ms length, with 100 ms overlap, was used to segment the signal. Information theory has shown that EMG segments with a length of 100–300 ms contain the highest information content. Furthermore, the optimal length for this specific task has been suggested to be between 100 and 300 ms [16].

**Fig. 2:**
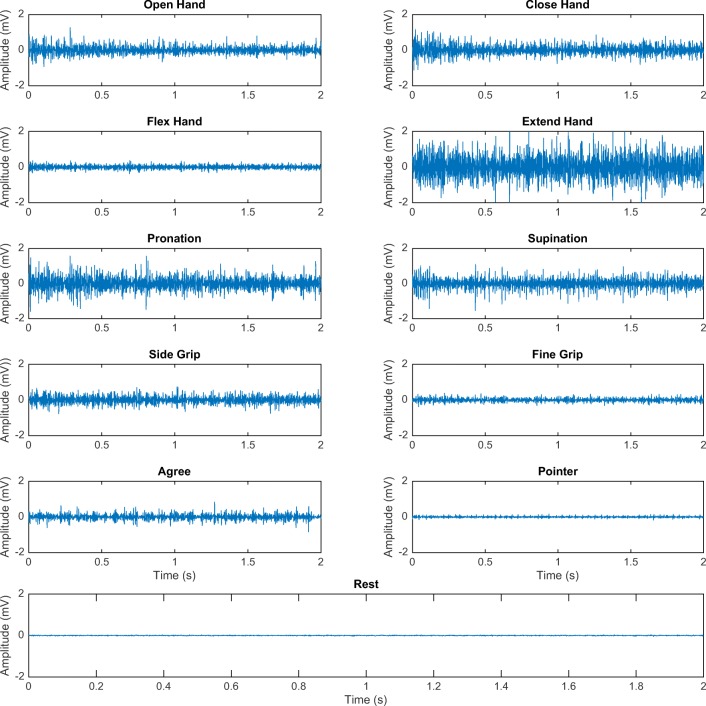
Two seconds of the EMG signal acquired from the first channel of one of the subjects during 10 hand motions and rest. mV, millivolt; s, second

Figure 2 shows the samples of the EMG signal acquired from the first channel of one of the subjects. The figure shows that the amplitude of the acquired EMG signals is different for each hand and finger motion. The EMG signal behaves similarly across all channels with different signal characteristics apparent for each movement class and channel. Signal features are calculated over each channel and window to be fed into the classifier for training and discrimination. The signal features over all channels will ideally result in vectors that can be cleanly separated in the feature space.

### Feature extraction

In total, 44 features in the time, frequency, and time-frequency domains were extracted from each window. The description of features extracted from EMG is introduced in this section. Thirty-six of these features are commonly used in this area (and were chosen by extensively reviewing the literature) and eight of them are new (which we would like to present their result). The eight new features, while not extensively reviewed for EMG processing, have shown initial promise in unpublished preliminary experiments.

#### Time domain features

TD features are extracted directly from pre-processed EMG without any transformation; therefore, they have low computational cost and are easy to implement [7]. This study extracted 25 features in TD from the surface EMG signals. Mean of peak values and mean firing velocity have not been evaluated for this purpose before.

1. Mean absolute value (MAV)

MAV is obtained by averaging the absolute value of the EMG signal in a window [4]. A large increase occurs in the value of this feature at onset and remains high during the contraction [3] and can be defined as [4]:

1 $$ \mathrm{MAV}=\frac{1}{N}\sum \limits_{i=1}^N\left|{x}_i\right| $$

where *x*_*i*_ is the EMG data and *N* is the number of samples in each time window (2000 Hz × 0.2 s = 400 samples) [3].

2) Standard deviation (STD)

STD represents the difference between each sample of EMG and its mean value [17] and is defined as:

2 $$ \mathrm{STD}={\left[\frac{1}{N-1}\sum \limits_{i=1}^N{\left({x}_i-\overline{x}\right)}^2\right]}^{\raisebox{1ex}{$1$}\!\left/ \!\raisebox{-1ex}{$2$}\right.} $$

where $\overline{x}$ represents the mean value of the EMG signal in a segment [18].

3) Variance (Var)

Variance represents the power of the EMG signal and helps to determine onset and contraction [3]. It can be obtained as [3, 19]:

3 $$ \mathrm{Var}=\frac{1}{N-1}\sum \limits_{i=1}^N{x}_i^2 $$

4) Waveform length

Waveform length of the signal gives information about complexity of the signal in a window by summing the numerical derivative of the sample window and can be calculated by [20]:

4 $$ \mathrm{WL}=\sum \limits_{i=1}^N\left(\left|{x}_i-{x}_{i-1}\right|\right) $$

5) Zero crossing (ZC)

ZC counts the number of times that the sign of the amplitude of the signal changes. It is calculated as [21]:

5 $$ \mathrm{ZC}=\sum \limits_{i=1}^N\operatorname{sgn}\left(-{x}_i{x}_{i-1}\right), $$

$$ \operatorname{sgn}(x)=\left\{\begin{array}{cc}1& \mathrm{if}\kern0.5em x>0\\ {}0& \mathrm{otherwise}\end{array}\right. $$

6) Number of peaks (NP)

Number of peaks is the number of values that are higher than their RMS value. RMS is calculated using Eq. (6) [22].

6 $$ \mathrm{RMS}=\sqrt{\frac{\sum \limits_{i=1}^N{\left|{x}_i\right|}^2}{N}} $$

7) Mean of peak values (MPV)

Mean of peak values is the average of the peak values that have been found in Section 6 [23].

8) Mean firing velocity (MFV)

Mean firing velocity is the difference or velocity of the peak values found in Section 6 [23].

9) Slope sign changes (SSC)

SSC represents the frequency properties of the EMG signal and it counts the number of times the slope of the EMG signal in a time window changes sign [6]. This can be defined as [13]:

7 $$ \mathrm{SSC}=\sum \limits_{i=2}^{N-1}f\left[\left({x}_i-{x}_{i-1}\right)\times \left({x}_i-{x}_{i+1}\right)\right] $$

$$ f(x)=\left\{\begin{array}{cc}1& \mathrm{if}\kern0.5em x>\mathrm{threshold}\\ {}0& \mathrm{otherwise}\end{array}\right. $$

10) Correlation coefficient

The Pearson correlation coefficient of all pairs of EMG channels in a time window shows the linear relationship between two samples of the EMG signal [24] and is defined as [25]:

8 $$ \mathrm{Cor}\left(x,y\right)=\frac{\left|\sum \limits_{i=1}^N\left({x}_i-\overline{x}\right)\left({y}_i-\overline{y}\right)\right|}{\sqrt{\sum \limits_{i=1}^N{\left({x}_i-\overline{x}\right)}^2\sum \limits_{i=1}^N{\left({y}_i-\overline{y}\right)}^2}} $$

where *x*_i_ and *y*_i_ are the EMG data of different channels.

11) Difference absolute mean value (DAMV)

DAMV is calculated as follows [15]:

9 $$ \mathrm{DAMV}=\frac{1}{N}\sum \limits_{i=1}^{N-1}\left|{x}_{i+1}-{x}_i\right| $$

12) Fractal dimension (FDim)

Fractal dimension of the EMG measures the strength of muscle activity and presents information about the active muscle (such as size and complexity) [19]. It is calculated as follows:

10 $$ \mathrm{FDim}(k)=\left\{\left(\sum \limits_{i=1}^{\left[\frac{N}{k}\right]}\left|X(ik)-X\left(\left(i-1\right)\ast k\right)\right|\right)\frac{N-1}{N}\right\}/k $$

where *N* is the sample window length and *k* is the time-step.

13) Maximum fractal length (MFL)

The maximum fractal length of the EMG measures the strength of muscle contraction specifically low-level muscle activation [19]. The definitions of this feature are expressed as follows [26]:

11 $$ \mathrm{MFL}={\log}_{10}\left(\sqrt{\sum \limits_{i=1}^{N-1}{\left({x}_{i+1}-{x}_i\right)}^2}\right) $$

14) Higuchi’s fractal dimension (HFD)

Higuchi’s fractal dimension is one of the most popular techniques that has shown good performance in calculating the fractal dimension. It can be calculated as follows [26]:

12 $$ \mathrm{HFD}=\frac{\log_{10}\left( FDim(1)\right)-{\log}_{10}\left( FDim(10)\right)}{\log_{10}(10)-{\log}_{10}(1)} $$

15) Skewness (Skew)

The Skewness describes asymmetry in a statistical distribution around the mean value and is calculated as [18]:

13 $$ \mathrm{Skew}=\frac{M_3}{M{}_2\sqrt{M{}_2}} $$

14 $$ {M}_k=\frac{1}{N}\sum \limits_{i=1}^N{\left({x}_i-\overline{x}\right)}^k $$

16) Integrated absolute value (IAV)

The integral of absolute value is a summation of absolute values of the EMG signal in a time window of *N* samples, which is given by [27]:

15 $$ \mathrm{IAV}=\sum \limits_{i=1}^N\left|{x}_i\right| $$

17) Hjorth mobility parameter (HMob)

Three parameters were introduced by Hjorth [28]: activity, mobility, and complexity (HCom). The activity parameter is the variance of the signal that was described previously in Section 3. The mobility parameter is proportional to the standard deviation of the power spectrum and is defined as [29, 30]:

16 $$ \mathrm{HMob}=\sqrt{\frac{\mathrm{Var}\left(\frac{dx(t)}{dt}\right)}{\mathrm{Var}\left(x(t)\right)}} $$

18) Hjorth complexity parameter

Complexity is the third feature of the Hjorth parameters which compares the similarity of the shape of a signal with a pure sine wave and can be calculated as [29, 30]:

17 $$ \mathrm{HCob}=\frac{\mathrm{Mobility}\left( dx(t)/ dt\right)}{\mathrm{Mobility}\left(x(t)\right)} $$

19) Multi-channel energy ratio

The absolute energy of the EMG signal from one channel is given by [24]:

18 $$ {E}_j=\sum \limits_{i=1}^N{x}_i^2 $$

where *j* is the *j*th channel of the EMG. The energy ratio of the *j*th channel to the *k*th channel signals is calculated by [24]:

19 $$ {\mathrm{ER}}_{jk}^{\ast }=\frac{E_j}{E_k},\kern1em j=2,...,M-1,\kern1em K=j+1,...M. $$

The normalization of the energy ratio (${\mathrm{ER}}_{jk}^{\ast }$) with respect to the first channel EMG signal is defined as [24]:

20 $$ {\mathrm{ER}}_{jk}=\frac{E_j\times {E}_1}{E_k^2} $$

20) Difference absolute standard deviation value (DASDV)

DASDV is a standard deviation value of the difference between the adjacent samples, which is calculated by [15, 22]:

21 $$ \mathrm{DASDV}=\sqrt{\frac{1}{N-1}\sum \limits_{i=1}^{N-1}{\left({x}_{i+1}-{x}_i\right)}^2} $$

21) Willison amplitude (WAM)

WAM in a time window counts the number of times the absolute value of the difference between two adjacent samples exceeds a predefined threshold. Its value indicates the muscle contraction levels [3].

22 $$ \mathrm{WAM}=\sum \limits_{i=1}^Nf\left(\left|{x}_i-{x}_{i+1}\right|\right), $$

$$ f(x)=\left\{\begin{array}{cc}1& \mathrm{if}\kern0.5em x>\mathrm{threshold}\\ {}0& \mathrm{otherwise}\end{array}\right. $$

22) Mean absolute value slope (MAVS)

Mean absolute value slope is the difference between mean absolute value of adjacent time windows and is defined as [4, 20]:

23 $$ \mathrm{MAVS}={\mathrm{MAV}}_{i+1}-{\mathrm{MAV}}_i\kern1em \mathrm{for}\kern1em i=1,...,I-1 $$

23) Kurtosis (Kurt)

Kurtosis describes the shape of a statistical distribution compared with the normal distribution and is defined as [18]:

24 $$ \mathrm{Kurt}=\frac{M_4}{M_2{M}_2} $$

where *M*is defined in Eq. (14).

24) Percentile (Perc)

The 75th percentile of the signal distribution is given by [18]:

25 $$ \operatorname{card}\left\{{x}_i/{x}_i<\mathrm{Perc}75\right\}=\frac{75N}{100} $$

where card is the number of elements in the set.

25) Histogram (Hist)

EMG histogram is an extended version of the ZC and WAM features that sorts the samples of the EMG signal from its minimum value to the maximum, segments the sorted values into several equally spaced frames, and returns the number of samples in each segment [21].

#### Frequency domain features

FD features are usually statistical properties of power spectral density (PSD) of EMG signals [7]. We defined eight frequency domain features as follows, three of which (waveform length, mean of peaks, and standard deviation of peaks) have not been investigated before. For all FD features, the fast Fourier transform was applied to the TD signal without padding.

#### Waveform length (WL)

WL in the frequency domain is calculated using Eq. (4) over the magnitude of the fast Fourier transform [23]. This also gives an estimate of the signal complexity, but in the frequency domain.

#### Mean frequency (MNF)

MNF is an average frequency value that can be calculated as [6]:

26 $$ \mathrm{MNF}=\frac{\sum \limits_{j=1}^M{f}_j{p}_j}{\sum \limits_{j=1}^M{p}_j} $$

where *f*_*j*_ is the frequency variable at frequency bin *j*, *p*_*j*_ is the power spectrum of the EMG signal at frequency bin *j*, and M is length of the frequency bin.

#### Median frequency (MDF)

MDF is a frequency at which the EMG power spectrum is divided into two parts with equal amplitude; it can be defined as [6]:

27 $$ \mathrm{MDF}=\frac{1}{2}\sum \limits_{j=1}^M{p}_j $$

#### Mean of peaks (MPK)

Similar to the MPV, the average of the peak values exceeding the RMS value of the EMG signal in frequency domain is calculated to form the MPK feature.

#### Standard deviation of peaks

After applying FFT to all channels of the EMG signals in the time domain, the STD of the peak values of the EMG signal in frequency domain is calculated to obtain the standard deviation of peaks (STDPK).

#### Frequency ratio (FR)

FR provides information to differentiate between contraction and relaxation of muscle and it is defined as the ratio of power spectrum at low-frequency band and high-frequency band. [4]:

28 $$ \mathrm{FR}=\frac{\sum \limits_{\mathrm{low}-\mathrm{frequency}\kern0.5em \mathrm{band}}{p}_j}{\sum \limits_{\begin{array}{cc}\mathrm{high}-\mathrm{frequency}& \mathrm{band}\end{array}}{p}_j} $$

The frequency bands are decided through the experiments. For example, Han et al. in 2000 [31] considered 30–250 Hz as the low-frequency band and 250–1000 Hz as the high-frequency band. In this study, we applied a band-pass filter with a bandwidth of 10–500 Hz as a pre-processor; therefore, the low- and high-frequency bands were changed to 10–250 and 250–500 Hz, respectively.

#### Peak frequency (PKF)

Peak frequency is the frequency of the maximum power. It is defined as [32]:

29 $$ \mathrm{PKF}=\max \left({p}_j\right)\kern1.5em j=1,\dots, M. $$

#### Frequency energy (FE)

To obtain the frequency energy feature, after computing the FFT for each sample, the FFT amplitude is squared. Then, the summation of the energy of all channels into 10 Hz bins is calculated [33].

#### Time-frequency domain features

A discrete wavelet transform (DWT) using fourth-order Coiflet mother wavelet and a wavelet packet transform using a fifth-order Symmlet mother wavelet (as recommended in [34]) were applied to the time domain signal to create a four-level wavelet decomposition (as recommended in [35]) of the EMG signal. Then, a total of 11 features were extracted from the wavelet and wavelet packet coefficients in the fourth (last) level. WL, mean, and MAV of wavelet coefficients have not been used before.

#### Standard deviation of wavelet coefficients

After applying DWT to each segment of the EMG signal, standard deviation of the wavelet coefficients in the last level was calculated [35].

#### Variance of wavelet coefficients

First, each segment of the EMG signal was decomposed using DWT; then, variance of the wavelet coefficients in the last level was calculated [35].

#### Waveform length of wavelet coefficients

After applying wavelet transform to each window of the EMG signal, waveform length of the wavelet coefficients in the last level was calculated.

#### Energy of wavelet coefficients

The EMG signal is decomposed by wavelet transform into four levels; then, the energy of the wavelet coefficients is determined in the last level as components of the feature vector [36, 37].

#### Maximum absolute value of wavelet coefficients

The maximum absolute value (MaxAV) of the wavelet coefficients in the last level was calculated as the feature vector of EMG signals [38].

#### Zero crossing of wavelet coefficients

After decomposing the EMG signal using DWT, the number of ZC of the wavelet coefficients in the last level is evaluated [36, 39].

#### Mean of wavelet coefficients

The EMG signal was decomposed by DWT into four levels; then, the mean of the wavelet coefficients in the last level was calculated.

#### Mean absolute value of wavelet coefficients

After decomposing the EMG signal using DWT into four levels, the mean absolute value of the wavelet coefficients in the last level was calculated.

#### Logarithmic RMS of wavelet packet coefficients

After applying the wavelet packet transform (WPT) and decomposing the EMG into four levels, the logarithmic RMS (LogRMS) of the coefficient in the last subspace was calculated [40].

#### Relative energy of wavelet packet coefficients

After the EMG had been decomposed by WPT, the relative energy (RE) of the coefficients in every subspace was employed as the signal feature set. The energy in each subspace can be computed as follows [41]:

30 $$ {E}_{j,p}=\sum \limits_i{\left|{w}_j^p(i)\right|}^2 $$

where *w* is the matrix of wavelet packet coefficients and *p* and *j* are the indexes of subspace and decomposition level, respectively. The total energy of the signal is given by:

31 $$ {\mathrm{TE}}_j=\sum \limits_p{E}_{j,p} $$

The relative energy of the signal in each subspace is:

32 $$ {\mathrm{RE}}_{j,p}=\raisebox{1ex}{${E}_{j,p}$}\!\left/ \!\raisebox{-1ex}{${\mathrm{TE}}_j$}\right. $$

#### Normalized logarithmic energy of wavelet packet coefficients

WPT was applied to the EMG signals to generate wavelet coefficients up to a level *j* decomposition. The logarithmic operator was then applied to the accumulation of the squares of the coefficients divided by the number of coefficients (*N*) in the subspace. Normalized logarithmic energy (NLE) is defined as follows [40]:

33 $$ {\mathrm{NLE}}_{j,p}=\log \left(\frac{\sum \limits_i{\left({w}_j^p(i)\right)}^2}{\raisebox{1ex}{$N$}\!\left/ \!\raisebox{-1ex}{${2}^j$}\right.}\right) $$

### Dimensionality reduction

To increase the classification performance, dimensionality reduction of the feature set is often necessary [13]. In this study, two types of dimensionality reduction were applied: feature selection (forward feature selection and backward feature elimination) and feature projection (PCA). Forward feature selection and backward feature elimination consist of adding features one by one to the feature set. If an added feature produced higher accuracy rate, it would stay in the feature set; otherwise it would be removed. Once all features were evaluated, features in the obtained feature set were removed in inverted order if their subtraction did not negatively affect accuracy. The 25 features in TD, the eight features in FD, and the 11 features in TFD were considered as three different feature sets. Then the feature selection and PCA were employed to reduce the dimensionality of the aforementioned feature sets.

### Classification

The resulting feature vectors corresponding to 11 movements were then fed into the following classifiers: LDA, k-nearest neighbor (KNN), decision tree (DT), maximum likelihood estimation (MLE), SVM, and multilayer perceptron (MLP). LDA, KNN, SVM, and MLP were chosen by reviewing state of the art; DT and MLE have been extensively used in different areas such as gait measurement and speech recognition [42]; however, there is no enough investigation on their performance on hand gesture recognition. First, the data of the 11 hand motions were used as a learning data set for the classifiers. Then, another data set of similar motions (from the same experiment of the same subject) was applied to the classifiers as test data for making decision regarding the kind of motion.

The results of the classifiers were performed by a 10-fold cross-validation in each subject and the classification accuracy was computed as an average accuracy based on the results from cross-validation testing of all subjects. The best performance of a feature is approached when the classification accuracy reaches its highest value [6].

#### Linear discriminant analysis

LDA is a simple and efficient classifier that has been used due to its high performance in classification of EMG signals, the robustness in long-term effect usage, and the low computational cost [6]. Discriminant analysis algorithm (type: linear) in MATLAB 2015b was used to classify the 11 hand motions.

#### K-nearest neighbor

KNN is a simple machine learning algorithm with a low training time that utilizes a distance measure relative to the k-nearest neighbors of a point to assign an unknown event to a given class [9, 21, 43]. In this study, Euclidean distance was chosen as the distance metric. In choosing K in the KNN classifier, it was observed that a larger value of K decreases the classification accuracy. We carried out our experiments with different values of K: 1 to 10, 40, and 100; K = 2 eventually provided the highest classification accuracy.

#### Decision tree

DT uses a set of comparisons of features extracted from physiological signals to classify the unknown input [42, 44]. To perform the DT classifier, the available algorithm in MATLAB 2015b was used.

#### Maximum likelihood estimation

The MLE is used for parameter estimation in statistics [15, 45]. For Gaussian inputs, these parameters are the mean and the covariance of the probability density function [15, 45]. In this study, the extracted features from the EMG signals were applied to the MLE in order to estimate the optimal parameters (one Gaussian model per class), and a group of the motion data was then determined.

#### Support vector machine

SVM is an increasingly popular machine learning tool that uses kernels to map data into separable hyper-planes [2, 9]. In this study, the LIBSVM library (C-SVC) was used to classify 11 different hand motions and the Kernel type was set to a Gaussian radial basis function [46]. In order to optimize the decoding performance and maximize correct classification, all parameters (such as cost and gamma in the kernel function) were set with a grid-search procedure.

#### Multilayer perceptron

Feedforward multilayer perceptron with two hidden layers of 16 neurons and 11 output neurons each (one neuron per movement) was used for classification. MLP can be implemented with more neurons and hidden layers, but doing so limits the ability to translate the results to real-time embedded systems useful for prosthetic control. The transfer function for the hidden layers is tan-sigmoid. The MLP was trained using a MATLAB’s Bayesian Regularization algorithm to prevent overtraining. The learning rate and momentum were 0.1.

### Feature and classification evaluation

All combinations of features and classifiers were used to perform the initial evaluation. We then investigated the accuracy rate of different combinations of features in each feature set (TD, FD, TFD) by applying the dimensionality reduction methods.

### Data analysis

Classification accuracy (acc) was computed as the average class-wise accuracy, defined as:

34 $$ \mathrm{ac}{\mathrm{c}}_i=\frac{\mathrm{T}{\mathrm{P}}_i+\mathrm{T}{\mathrm{N}}_i}{\mathrm{T}{\mathrm{P}}_i+\mathrm{T}{\mathrm{N}}_i+\mathrm{F}{\mathrm{P}}_i+\mathrm{F}{\mathrm{N}}_i} $$

where *i* is the class index and *TP*, *TN*, *FP*, and *FN* are true positive, true negative, false positive, and false negative predictions, respectively. Accuracy averaged over all classes and further averaged over cross-validation results.

Performance matrixes between movements (intended and detected movements) were also used to better visualize and compare the performance of different algorithms in detecting different movements. Each row of the matrix represents the intended movements while each column represents the detected movements.

Another important issue for motion recognition is the time consumption for training and classification. Therefore, the elapsed time of each classifier in combination with features and feature sets that obtained better accuracy rates in each domain was calculated in second (s).

**Fig. 3:**
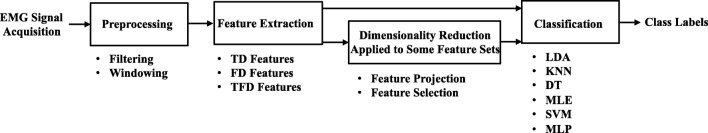
Flow chart of the processing stages of different hand and finger movement recognition

The processing stages in this study for decoding hand and finger movements are presented in Fig. 3.

To compare the results, we also applied all the classifiers to the Hudgins feature set (a set of TD features including MAV, WL, SSC, ZC, and DAMV introduced by Hudgins et al. [10]). One-way analysis of variances (ANOVA) using general linear model procedure of SAS software (SAS Institute Inc. 2004) was carried out by analyzing the parameters as a completely randomized design to find statistically significant differences among the obtained accuracies by each feature and feature set. Duncan’s multiple range test was used to test the significance of the difference between means. All significances were declared at *p* < 0.0001.

## Results

The average classification accuracy and standard deviation of different feature/classifier combinations across the 20 subjects are shown in Tables 1, 2, 4, and 6. The least significant difference tests [47], which were used to determine significant differences among obtained accuracy rates, are also presented in Tables 1, 2, 4, and 6.

**Table 1:** The average classification accuracy and standard deviation of single TD feature/classifier combinations across 20 subjects

TDF		Average classification accuracy (%) ± STD					
		LDA	KNN	DT	MLE	SVM	MLP
1	MAV	84.65^c^ ± 7.73	93.17^ab^ ± 4.60	90.05^abc^ ± 4.92	89.93^bcd^ ± 6.21	84.02^bc^ ± 8.47	91.14^bcdef^ ± 4.69
2	STD	84.66^c^ ± 7.95	93.52^a^ ± 3.56	89.75^abcd^ ± 5.01	89.45^bcd^ ± 6.05	84.62^bc^ ± 7.62	91.00^cdef^ ± 4.20
3	Var	71.21^f^ ± 11.35	90.42^abc^ ± 4.94	89.95^abc^ ± 4.68	79.19^gh^ ± 8.40	72.83^e^ ± 10.14	90.51^cdefg^ ± 4.11
4	WL	84.71^c^ ± 7.43	93.16^ab^ ± 4.38	90.18^abc^ ± 5.47	90.44^bc^ ± 5.88	84.86^bc^ ± 8.08	91.21^bcde^ ± 4.69
5	ZC	55.48^mn^ ± 8.45	47.26^lm^ ± 9.63	47.69^pq^ ± 8.69	53.70^nop^ ± 8.10	49.50^mn^ ± 8.14	52.12^rs^ ± 8.55
6	NP	57.92^klmn^ ± 8.53	51.43^jkl^ ± 10.42	51.34^op^ ± 8.80	55.44^mno^ ± 8.29	54.55^ijkl^ ± 9.20	55.81^pqr^ ± 8.90
7	MPV	83.49^cd^ ± 7.81	92.29^abc^ ± 4.36	88.83^abcd^ ± 5.01	87.81^bcde^ ± 6.64	84.14^bc^ ± 7.11	89.85^defg^ ± 4.69
8	MFV	19.97^r^ ± 1.99	19.65^q^ ± 1.69	18.43^v^ ± 1.68	20.41^tu^ ± 1.64	19.71^s^ ± 1.94	19.93^w^ ± 1.79
9	SSC	59.19^jklm^ ± 9.08	50.01^klm^ ± 9.61	51.39^op^ ± 9.56	58.08^lmno^ ± 8.97	56.61^hij^ ± 8.79	55.42^qr^ ± 9.28
10	Cor	62.89^hij^ ± 9.07	57.46^ghi^ ± 10.05	52.98^no^ ± 9.56	61.43^kl^ ± 9.35	58.18^ghi^ ± 9.43	54.98^rs^ ± 10.51
11	DAMV	84.85^c^ ± 7.45	93.29^ab^ ± 4.50	90.29^abc^ ± 5.40	90.42^bc^ ± 5.84	84.01^bc^ ± 8.11	91.18^bcdef^ ± 4.72
12	FDim	37.41^pq^ ± 8.30	34.31^n^ ± 8.21	34.14^rst^ ± 8.99	34.73^r^ ± 8.45	33.73^pq^ ± 7.11	37.64^uv^ ± 8.99
13	MFL	84.88^c^ ± 7.47	93.95^a^ ± 4.41	90.16^abc^ ± 5.50	90.4^bc^ ± 5.98	84.24^bc^ ± 7.73	91.46^abcde^ ± 4.41
14	HFD	41.07^op^ ± 7.29	33.42^no^ ± 6.84	33.33^rst^ ± 6.75	40.17^q^ ± 7.20	38.36^op^ ± 6.27	38.15^uv^ ± 7.24
15	Skew	35.84^q^ ± 6.88	28.90^op^ ± 5.93	29.77^tu^ ± 6.52	33.78^r^ ± 6.40	27.40^r^ ± 5.51	34.08^v^ ± 6.46
16	IAV	84.57^c^ ± 7.75	93.23^ab^ ± 4.49	90.01^abc^ ± 5.13	89.97^bc^ ± 6.04	83.97^bc^ ± 8.08	91.62^abcde^ ± 4.46
17	HMob	53.78^n^ ± 10.05	45.60^m^ ± 11.24	46.12^q^ ± 10.71	53.27^op^ ± 10.56	48.73^mn^ ± 10.46	50.61^s^ ± 9.52
18	HCom	68.72^fg^ ± 8.71	66.18^f^ ± 11.38	63.02^jk^ ± 10.03	67.45^ij^ ± 8.74	59.74^fgh^ ± 11.63	66.63^l^ ± 9.18
19	ER	57.85^lmn^ ± 9.55	76.47^e^ ± 5.34	80.84^gh^ ± 6.10	69.34^i^ ± 9.73	54.87^hijkl^ ± 8.46	83.14^ij^ ± 5.16
20	DASDV	83.95^cd^ ± 7.95	92.26^abc^ ± 4.50	88.94^abcd^ ± 5.31	88.25^bcde^ ± 5.98	85.01^bc^ ± 7.44	89.75^defg^ ± 4.29
21	WAM	71.11^f^ ± 14.75	75.24^e^ ± 14.43	78.82^h^ ± 11.28	60.07^klm^ ± 28.44	77.40^de^ ± 13.88	79.57^jk^ ± 11.11
22	MAVS	11.52^s^ ± 2.89	32.82^no^ ± 5.67	32.21^st^ ± 5.56	26.23^s^ ± 3.68	12.45^t^ ± 1.40	18.52^w^ ± 5.10
23	Kurt	35.80^q^ ± 6.72	35.84^n^ ± 8.16	35.08^rs^ ± 8.09	32.76^r^ ± 6.58	29.97^qr^ ± 6.53	39.09^u^ ± 7.97
24	Perc	81.36^cde^ ± 8.16	89.60^abc^ ± 6.15	86.32^bcde^ ± 6.68	85.81^cdef^ ± 7.43	80.15^cd^ ± 8.82	87.73^efgh^ ± 5.38
25	Hist	42.04^op^ ± 6.97	46.42^m^ ± 5.81	34.35^rst^ ± 6.87	40.57^q^ ± 6.72	41.91^o^ ± 6.39	40.25^tu^ ± 7.67

^a,b^Means within a column not sharing a common superscript are significantly different (*p* < 0.0001)

**Table 2:** The average classification accuracy and standard deviation of TD feature set/classifier combinations across 20 subjects

TD feature sets	Average classification accuracy (%) ± STD					
	LDA	KNN	DT	MLE	SVM	MLP
FS	95.15^a^ ± 2.85	94.07^a^ ± 3.77	91.36^a^ ± 4.56	97.43^a^ ± 1.70	91.97^a^ ± 3.82	93.73^abcd^ ± 3.22
Hudgins feature set	89.90^b^ ± 5.23	88.68^bc^ ± 7.02	90.60^ab^ ± 5.62	91.31^b^ ± 5.12	86.28^b^ ± 7.53	91.64^abcde^ ± 4.26
Applying PCA to 25 TD features	96.20^a^ ± 2.44	92.54^abc^ ± 5.02	79.66^h^ ± 10.49	83.81^efg^ ± 7.09	94.73^a^ ± 3.20	94.39^abc^ ± 3.18

^a,b^Means within a column not sharing a common superscript are significantly different (*p* < 0.0001)

### TD feature result

Of the 25 TD features, nine features including one of the new features (MPV)—MAV, STD, WL, MPV, DAMV, MFL, IAV, DASDV, and Perc—showed the best performance for the six classifiers. The KNN and MLP classifiers showed numerically higher accuracy rates than the rest, but no statistical significance was observed. Combining the KNN classifier with each of the aforementioned features produced accuracy rates with averages of 93.17%, 93.52%, 93.16%, 92.29%, 93.29%, 93.95%, 93.23%, 92.26%, and 89.60%, respectively. The MAVS/LDA combination obtained the lowest rate, with an average of 11.52%.

For the feature set evaluation using the forward feature selection and backward feature elimination, Var, WL, Cor, HMob, and HCom were found to improve the results, referred to here as FS, our proposed feature set. The highest accuracy rate of each investigated classifier in this study (LDA, KNN, DT, MLE, SVM, and MLP) in combination with the 25 TD features was 84.88%, 93.95%, 90.29%, 90.44%, 85.01%, and 91.62%, respectively (Table 1). Combining TD features and proposing the new feature set (FS) increased the accuracy rates of LDA, MLE, and SVM classifiers to 95.15%, 97.43%, and 91.97%, respectively (Table 2), which was statistically significant (*p* < 0.0001). There was also a numerical increase in the accuracy rates of KNN, DT, and MLP classifiers, to 94.07%, 91.36%, and 93.73%, respectively; however, this increase was not statistically significant.

The Hudgins feature set in Table 2 showed significantly lower accuracy rates (*p* < 0.0001) than FS for LDA, KNN, MLE, and SVM classifiers. The highest accuracy rate for the Hudgins feature set was obtained by the MLP classifier, with an average of 91.64%. PCA was also applied to all 25 TD features as a feature set to decrease the dimension of the feature set from 133 to 20, a number experimentally found to offer a reasonable trade-off between accuracy and complexity. The DT and MLE classifiers, using the FS set, showed significantly (*p* < 0.0001) higher accuracy than the TD features/PCA combination. For the LDA, KNN, SVM, and MLP classifiers, there was no statistically significant difference between the rates of TD features/PCA combination and our proposed feature set. However, the accuracy rates of LDA, SVM, and MLP classifiers in conjunction with TD features/PCA combination were numerically higher than that of the FS set with an average of 96.20%, 94.73%, and 94.39%, respectively.

Table 3 shows the performance matrix between the movements (the intended movements and the detected movements) for the FS/MLE combination that obtained the highest classification accuracy among the TD features. As presented in Table 3, flex hand and extend hand were easy to detect as they obtained the highest classification accuracies (above 99%). However, there was confusion between flex hand and open hand, pronation, and others when using the FS/MLE combination. There was also some difficulty discriminating extend hand from open hand, close hand, side grip, and fine grip. The lowest classification accuracy with an average of 94.43% was obtained by fine grip indicating that this movement was the most difficult to detect. It was confused most often (1.88% of the time) with side grip.

**Table 3:** The performance matrix between the movements (the intended movements and the detected movements); numbers are average classification accuracy (%) obtained by the FS/MLE combination across 20 subjects

	Detected movements											
		OH	CH	FH	EH	PR	SU	SG	FG	AG	PO	RST
Intended movements	OH	98.36	0.16	0.08	0	0.08	0.25	0.16	0.08	0.25	0.41	0.16
	CH	0.25	97.79	0.08	0.08	0	0	0.66	0	0.66	0.33	0.16
	FH	0.16	0,0	99.26	0	0.33	0	0	0	0	0.16	0.08
	EH	0.16	0.08	0	99.51	0	0	0.08	0.16	0	0	0
	PR	0.49	0	0.16	0	98.93	0.08	0	0.08	0.08	0.16	0
	SU	0.33	0.08	0	0.16	0.08	98.77	0.16	0.08	0.16	0.16	0
	SG	0.74	0.49	0	0.08	0.33	0	95.33	0.90	1.07	1.07	0
	FG	0.66	0.41	0.08	0.16	1.23	0.25	1.88	94.43	0.33	0.57	0
	AG	0.33	0.81	0.08	0	0.08	0.33	0.66	0.16	95.90	1.64	0
	PO	0.25	0.82	0.25	0.08	0.25	0.33	0.33	0.16	1.31	96.23	0
	RST	0.49	0.25	0.08	0	0.33	0	0.90	0.16	0.41	0.16	97.21
	Average = 97.43%											

*OH* open hand, *CH* close hand, *FH* flex hand, *EH* extend hand, *PR* pronation, *SU* supination, *SG* side grip, *FG* fine grip, *AG* agree, *PO* pointer, *RST* rest

### FD feature results

Most of the FD features obtained low accuracy rates; among the eight FD features tested, WL and our proposed feature, MPK, showed only marginally better performance. The highest rate was obtained by FE/KNN combination with an average of 90.02% (Table 4). The combination of the FE feature and the MLE classifier obtained the lowest accuracy rate among the FD features, with an average of 16.80%.

**Table 4:** The average classification accuracy and standard deviation of single FD feature/classifier combinations across 20 subjects

FDF		Average classification accuracy (%) ± STD					
		LDA	KNN	DT	MLE	SVM	MLP
1	WL	82.42^cde^ ± 7.78	88.27^c^ ± 5.86	85.81^cdef^ ± 6.08	86.61^bcde^ ± 6.53	81.14^cd^ ± 7.64	86.97^fghi^ ± 4.95
2	MNF	66.55^fgh^ ± 8.97	64.23^f^ ± 11.04	61.22^jk^ ± 10.46	64.59^ijk^ ± 8.47	57.81^ghij^ ± 11.63	64.25^lm^ ± 9.44
3	MDF	61.10^ijkl^ ± 8.13	57.58^ghi^ ± 8.50	59.18^lmn^ ± 8.65	58.74^lmn^ ± 7.75	50.06^lmn^ ± 11.07	59.87^nop^ ± 7.71
4	MPK	80.80^cde^ ± 7.75	87.92^c^ ± 6.37	85.14^defg^ ± 6.73	84.82^def^ ± 7.35	80.94^cd^ ± 8.23	86.39^ghi^ ± 5.60
5	STDPK	42.55^o^ ± 5.91	36.33^n^ ± 6.64	37.90^r^ ± 6.98	37.76^rq^ ± 4.88	33.77^pq^ ± 6.57	43.66^t^ ± 6.89
6	FR	61.27^ijkl^ ± 8.24	55.26^hij^ ± 10.13	54.61^mno^ ± 9.51	59.5^klm^ ± 8.12	53.24^jklm^ ± 8.76	59.01^opq^ ± 8.87
7	PKF	77.77^e^ ± 8.34	83.26^d^ ± 9.01	81.40^fgh^ ± 7.99	80.69^fgh^ ± 8.61	75.22^e^ ± 10.59	82.75^ij^ ± 7.15
8	FE	64.06^ghi^ ± 9.23	90.02^abc^ ± 6.41	82.52^efgh^ ± 7.87	16.80^u^ ± 6.76	62.55^fg^ ± 9.71	61.65^mno^ ± 10.35
Applying PCA to eight FDF		81.89^cde^ ± 8.37	90.78^abc^ ± 6.44	78.55^hi^ ± 9.42	18.99^tu^ ± 8.00	63.13^f^ ± 7.91	85.22^hi^ ± 5.07

^a,b^Means within a column not sharing a common superscript are significantly different (*p* < 0.0001)

Different combinations of the FD features were investigated using the forward feature selection and backward feature elimination; however, the results did not improve significantly. PCA was applied to the eight FD features to decrease the dimensionality of the feature set from 828 to 20. The accuracy rate of the KNN classifier showed a slight numerical increase, but the other classifiers obtained lower accuracy rates when employing the PCA (Table 4).

As illustrated in Table 5, the FE/KNN combination (the best performing combination among the FD features) showed the highest average accuracy of 98.52% for rest (no movement) with flex hand showing the next highest accuracy of 97.46%. Rest was confused with open hand and close hand by the FE/KNN combination the most, but each was less than 1% of the total predictions for the class. The lowest classification accuracy with an average of 84.43% was obtained by side grip. This movement was confused with all the movements to some extent except flex hand and extend hand; the most confusion happened with fine grip with an average prediction rate of 5.90%.

**Table 5:** The performance matrix between the movements (the intended movements and the detected movements); numbers are average classification accuracy (%) obtained by the FE/KNN combination across 20 subjects

	Detected movements											
		OH	CH	FH	EH	PR	SU	SG	FG	AG	PO	RST
Intended movements	OH	87.46	0.33	0	0.08	1.64	0.98	1.97	1.72	1.97	2.70	1.15
	CH	0.41	85.66	0	0.33	0.98	0.25	4.26	2.62	2.54	2.30	0.57
	FH	0.66	0.16	97.46	0	0.49	0	0.08	0.16	0	0.41	0.33
	EH	1.15	0.74	0	95.25	0	0	0.41	0.57	1.15	0	0
	PR	3.28	0.74	0.33	0	88.11	2.13	1.56	1.64	1.15	0.33	0
	SU	0.74	0.25	0	0	0.57	90.33	0.33	2.21	2.70	1.72	0.98
	SG	1.64	1.39	0	0	0.82	0.41	84.43	5.90	2.30	1.39	1.64
	FG	0.49	1.39	0	0	0.74	0.66	5.98	86.39	1.80	0.66	1.89
	AG	1.39	0.74	0	0	0	2.13	1.39	1.80	88.28	2.13	1.15
	PO	1.23	1.56	0	0	0.08	1.64	1.23	3.03	1.15	88.20	1.80
	RST	0.08	0.16	0	0	0	0	0	0	0	0	98.52
	Average = 90.0%											

*OH* open hand, *CH* close hand, *FH* flex hand, *EH* extend hand, *PR* pronation, *SU* supination, *SG* side grip, *FG* fine grip, *AG* agree, *PO* pointer, *RST* rest

### TFD feature result

Table 6 presents the classification accuracy of 11 different TFD features (the first eight features were extracted from the wavelet coefficients and the last three features were extracted from the wavelet packet coefficients). Afterwards, PCA was applied to all 11 TFD features as a set to decrease dimension of the feature set from 224 to 20. The wavelet transform features did not have good performance in decoding the 11 hand movements and the highest accuracy rate, with an average of 67.66%, was obtained with the WL/MLP combination (Table 6). The forward feature selection and backward feature elimination were applied to the TFD features; however, the results did not improve significantly.

**Table 6:** The average classification accuracy and standard deviation of single TFD feature/classifier combinations across 20 subjects

TFDF		Average classification accuracy (%) ± STD					
		LDA	KNN	DT	MLE	SVM	MLP
1	STD	61.0^ijkl^ ± 7.68	61.89^gf^ ± 9.62	62.44^jk^ ± 8.69	61.52^kl^ ± 7.60	55.38^hijk^ ± 8.12	66.35^l^ ± 6.81
2	Var	45.57^o^ ± 6.43	57.59^ghi^ ± 9.02	62.09^jk^ ± 8.30	50.12^p^ ± 7.05	47.98^n^ ± 6.57	64.57^lm^ ± 7.72
3	WL	62.57^hijk^ ± 8.08	64.57^f^ ± 9.22	63.55^j^ ± 8.67	62.40^jkl^ ± 7.95	55.94^hijk^ ± 7.45	67.66^l^ ± 7.62
4	Er	45.17^o^ ± 6.30	59.08^gh^ ± 9.82	60.54^jkl^ ± 8.59	49.38^p^ ± 7.35	48.49^mn^ ± 6.09	63.69^lmn^ ± 6.82
5	MaxAV	55.61^mn^ ± 7.13	58.42^gh^ ± 9.25	58.78^klm^ ± 8.67	56.29^mno^ ± 7.53	51.05^klmn^ ± 7.72	63.69^lmn^ ± 7.00
6	ZC	10.01^s^ ± 1.17	10.34^r^ ± 1.29	10.13^w^ ± 1.18	9.88^v^ ± 1.17	10.17^t^ ± 1.26	9.16^x^ ± 0.71
7	Mean	13.00^s^ ± 2.24	27.02^p^ ± 5.18	26.88^u^ ± 5.07	22.67^st^ ± 3.76	13.57^t^ ± 2.30	19.9^w^ ± 4.46
8	MAV	62.44^hijkl^ ± 7.99	61.72^fg^ ± 9.66	61.66^jk^ ± 9.37	62.38^jkl^ ± 8.29	58.94^fghi^ ± 8.78	65.94^l^ ± 8.13
9	LogRMS	95.24^a^ ± 3.63	93.35^a^ ± 4.94	86.63^bcde^ ± 6.04	76.15^h^ ± 9.02	93.51^a^ ± 5.51	95.47^a^ ± 3.51
10	RE	79.57^de^ ± 8.13	53.70^ijk^ ± 9.68	55.50^mno^ ± 10.39	37.24^rq^ ± 7.79	74.05^e^ ± 8.96	75.94^k^ ± 9.54
11	NLE	95.12^a^ ± 3.76	93.30^ab^ ± 5.00	86.47^bcde^ ± 6.29	76.20^h^ ± 9.56	93.81^a^ ± 5.29	95.24^ab^ ± 3.61
Applying PCA to 11 TFDF		94.87^a^ ± 3.97	90.33^abc^ ± 6.06	73.94^i^ ± 8.76	79.37^gh^ ± 9.65	93.33^a^ ± 5.55	85.39^hi^ ± 6.36

^a,b^Means within a column not sharing a common superscript are significantly different (*p* < 0.0001)

Among the TFD features, LogRMS and NLE showed the highest accuracy rates when applied to the LDA and MLP classifiers (with averages of above 95%). The above-mentioned features, as applied to the KNN and SVM classifiers, evolved into the second highest accuracy rates with averages of above 93% for KNN and SVM (Table 6). DT and MLE did not achieve good accuracy rates using TFD features. The LDA and SVM classifiers obtained the highest rate of 94.87% and 93.33% in conjunction with TFD features/PCA combination (almost the same result obtained by LogRMS and NLE features). The PCA dimensionality reduction improved the result of MLE classifier from 76.20 to 79.37%, which was not statistically significant. The results for the feature set analysis of TFD features using the feature selection method did not significantly improve the classification accuracy of any of the classifiers.

Table 7 illustrates the performance matrix between the movements (the intended movements and the detected movements) for the LogRMS/MLP combination which obtained the highest classification accuracy among the TFD features. Flex hand, extend hand, and pronation obtained the highest accuracy rates of above 98%. Flex hand was misclassified to some extent with all the movements except close hand and extend hand with the highest misclassification rate of 0.49% with pronation. The highest misclassification rate in detecting extend hand was found with fine grip at 0.49%. The lowest classification accuracy with an average of 91.31% was obtained by side grip. Side grip was confused with all movements except flex hand and extend hand; the highest misclassification rate was obtained by fine grip with an average accuracy of 3.61%.

**Table 7:** The performance matrix between the movements (the intended movements and the detected movements); numbers are average classification accuracy (%) obtained by the LogRMS/MLP combination across 20 subjects

	Detected movements											
		OH	CH	FH	EH	PR	SU	SG	FG	AG	PO	RST
Intended movements	OH	95.33	0.49	0.16	0.08	0.25	0.98	0.25	0.41	0.57	0.90	0.57
	CH	0.41	95.66	0	0.08	0	0.66	0.82	0.57	0.98	0.41	0.41
	FH	0.25	0	98.28	0	0.49	0.08	0.08	0.16	0.16	0.16	0.33
	EH	0.08	0.08	0	98.93	0.08	0.08	0.25	0.49	0	0	0
	PR	0.25	0	0.08	0	98.11	0.25	0.33	0.66	0.16	0.08	0.08
	SU	0.66	0.49	0.08	0	0.16	96.39	0.41	0.66	0.66	0.25	0.25
	SG	0.74	0.49	0	0	0.49	0.16	91.31	3.61	0.82	1.39	0.98
	FG	0	0.66	0.08	0.08	0.74	0.25	3.03	93.36	0.49	0.90	0.41
	AG	0.66	1.23	0.16	0	0.08	0.90	0.49	0.82	93.28	2.30	0.08
	PO	1.15	0.41	0.16	0	0.25	0.49	1.64	0.98	2.13	91.80	0.98
	RST	0.16	0.16	0.16	0	0	0	0.74	0.49	0	0.57	97.70
	Average = 95.47%											

*OH* open hand, *CH* close hand, *FH* flex hand, *EH* extend hand, *PR* pronation, *SU* supination, *SG* side grip, *FG* fine grip, *AG* agree, *PO* pointer, *RST* rest

### Processing time

The average values of offline training and testing times (proportional to the offline training and real-time classification delay, respectively) of the 20 subjects on the same feature extraction and classifiers are presented in Tables 8 and 9, respectively.

**Table 8:** Elapsed time in seconds (s) when training six classifiers with 11 different single and multiple features

Features	Elapsed time (s) for training					
	LDA	KNN	DT	MLE	SVM	MLP
MAV	0.092	0.016	0.021	0.008	0.060	5.207
STD	0.096	0.018	0.026	0.010	0.052	5.329
WL	0.095	0.018	0.024	0.011	0.055	6.540
DAMV	0.092	0.019	0.023	0.011	0.054	7.130
MFL	0.868	0.798	0.804	0.791	0.831	6.010
IAV	0.090	0.019	0.025	0.011	0.058	4.890
FS	0.198	0.128	0.140	0.121	0.191	7.189
Hudgins set	0.158	0.087	0.100	0.080	0.156	14.47
FE	0.666	0.358	0.753	3.067	1.345	3655.0
LogRMS	34.31	34.23	34.27	34.23	34.34	61.68
NLE	36.24	36.17	36.21	36.17	36.29	63.40

**Table 9:** Elapsed time in seconds (s) when testing six classifiers with 11 different single and multiple features

Features	Elapsed time (s) for testing					
	LDA	KNN	DT	MLE	SVM	MLP
MAV	0.074	0.009	0.007	0.021	0.010	0.021
STD	0.078	0.011	0.009	0.024	0.011	0.022
WL	0.077	0.012	0.010	0.023	0.013	0.024
DAMV	0.077	0.012	0.010	0.023	0.013	0.024
MFL	0.862	0.792	0.790	0.803	0.793	0.803
IAV	0.077	0.012	0.010	0.023	0.013	0.023
FS	0.185	0.121	0.119	0.136	0.123	0.131
Hudgins set	0.151	0.080	0.078	0.094	0.083	0.091
FE	0.503	0.298	0.297	1.457	0.433	0.342
LogRMS	34.30	34.22	34.22	34.24	34.23	34.23
NLE	36.23	36.16	36.16	36.18	36.17	36.17

The training process for KNN and MLE took less time than other classifiers, whereas MLP took the most time. The MAV/MLE combination was the fastest (0.008 s) and FE/MLP was the most time-consuming combination (3655.0 s) in training (Table 8). The testing process for KNN and DT was faster than the other classifiers, and LDA consistently took the most time. The MAV/DT combination had the fastest testing time, and NLE/LDA showed the slowest testing time (Table 9). Among the TD features, MFL was the most time-consuming. For FE, LogRMS, and NLE, the transformation of time domain signal to the frequency and time-frequency domains made these feature sets relatively time-consuming.

For LDA and SVM classifiers, FS, LogRMS, and NLE features provided the highest accuracy rates. However, LogRMS and NLE took much more time than FS for both training and testing (34.31 s vs. 0.198 s and 34.30 s vs. 0.185 s, respectively). For the KNN and DT classifiers, FS obtained the highest accuracy; however, MAV was the fastest for both testing and training. For MLE, the FS showed the highest accuracy rate, and the elapsed time for training and testing was 0.121 s and 0.136 s, respectively. For MLP, FS, LogRMS, and NLE showed the highest accuracy rates; however, FS was the fastest in both training and testing (7.189 s and 0.131 s, respectively).

## Discussion

The aim of the present study was to investigate configurations of signal features and classifiers to improve the accuracy and responsiveness of surface EMG-based motor decoding systems. To this purpose, four channels of surface EMG signal recorded from 20 healthy subjects during 11 different hand movements were selected from the BioPatRec database [14]. An investigation was then performed on the accuracy and responsiveness of 44 conventional and new features in combination with six different classifiers on a freely available database using a publicly available training and testing methodology. Many of the relative accuracy differences found in this work are corroborated with results from some of the existing literature [7, 11, 48], but the standardized and open pattern recognition framework used here provided results that are easily comparable with any other works that opt to use the same system.

The experimental results of the 25 TD features as presented in Table 1 showed that MAV, STD, WL, MPV, DAMV, MFL, IAV, and DASDV in combination with the KNN classifier obtained the highest accuracy rates (above 92%). Among these features, MFL was the most computationally expensive feature (Tables 8 and 9), limiting its usefulness in real-time systems.

Since the accuracy rate of the TD features was not satisfactory, we evaluated many different combinations of the 25 TD features and proposed an efficient feature set which provided statistically significant improvement in combination with LDA, MLE, and SVM classifiers. However, the elapsed training and testing time were slightly increased, but the authors still consider it acceptable for real-time applications. We then compared the result of our proposed feature set with that of a well-known feature set in the state of the art (Hudgins’ set). The results in Table 2 show that our proposed feature set outperformed the Hudgins’ TD feature set. The presented performance matrix for the FS/MLE combination in Table 3 shows that, among the movements, gross movements, like flex hand and extend hand, were easy to detect while fine grip was the most difficult. This is expected, as gross antagonist movements tend to generate highly separable features.

We also investigated the performance of eight FD features. However, they did not show sufficiently low delay in combination with any of the six classifiers under investigation. The highest accuracy was obtained with the FE/KNN combination with an accuracy of 90.2%, but the testing time for this combination was almost 0.3 s, over twice that of our proposed feature set. The provided performance matrix for this combination in Table 5 shows that, among the individual movements, rest (no movement) and flex hand obtained the highest accuracy, whereas side grip obtained the lowest. This indicates that the frequency content of system and environmental noise, which dominates the EMG recordings at rest, has significantly different frequency characteristics than EMG signals.

Among the 11 investigated TFD features, the features that were obtained from the wavelet packet coefficients (LogRMS and NLE) had the highest accuracy when used in combination with LDA and MLP classifiers (above 95%). However, the FD and TFD features were computationally expensive, since the TD EMG signal needs to be transformed to the frequency and time-frequency domains, respectively. The performance matrix in Table 7 illustrates that flex hand, extend hand, and pronation were the easiest to detect and side grip was the most difficult, which corroborates our previous conclusions.

PCA was also applied to the three sets of features (25 TD features, eight FD features and, 11 TFD features) in each domain as the dimensionality reduction method and the results in Tables 2, 4, and 6 show that, in some cases, the accuracy rate dropped. This indicates that the reduced feature set lost some useful information from the EMG signal. However, almost the same result was obtained with a lower dimension (133 vs. 20, 224 vs. 20, and 828 vs. 20) for some of the combinations such as FE/KNN and FD/PCA in combination with KNN, suggesting that some classifiers, like KNN, are more robust to the information loss.

Overall, a wide range of pattern recognition methods, some of which have been evaluated in different studies with varying methodologies, were gathered into one study with the same experimental setup to find efficient configurations, resulting in an efficient feature set that improves motion recognition accuracy while maintaining high responsiveness.

In future work, other promising alternative classifiers, like deep–learning algorithms and cascade classification schemes [49], will be used to decode individual hand movements and will be compared with conventional machine learning algorithms shown in this work. Improved parameter selection for the conventional machine learning will also be investigated. The data from other custom-collected datasets will also be used to observe the amount of deviations in the performance of algorithms and to enhance the statistical robustness of the comparisons. Optimal feature and classifier combinations will then be used in real-time tests, like the Target Achievement Control [50] or Motion Test [51], to corroborate the findings and translate them to clinically useful technologies.

## Conclusions

In this study, we investigated 44 EMG features and five combinations of such features, and used six classifiers to decode 11 human upper-limb movements. Even though the processing time and dimension for the TD features were faster and smaller than other features, recognition performance was found to be unsatisfactory. Therefore, a new feature set (FS) was proposed by combining different TD features, which offered statistically significant improvement (*p* < 0.0001) in the results, with the cost of a small increase in the elapsed time for both training and testing processes. Among the classifiers under investigation, KNN and MLP offered the best performance for time domain features. However, MLP took much longer to train. LDA and MLP showed higher accuracy than other classifiers when used in combination with wavelet packet features (LogRMS and NLE), but at the cost of training and testing time. LDA was much faster than MLP when training, but the reverse was true for testing. MLE and SVM obtained their highest rates with the TD and TFD features, but they showed unsatisfactory performance in combination with FD features. The FS/MLE combination obtained the highest accuracy rate (above 97%) among the FD features. However, the elapsed time for the training and testing was 0.358 s and 0.298 s, respectively, making this combination unsuitable for real-time pattern recognition. PCA was also applied to the three feature sets in TD, FD, and TFD, and the results indicated that applying PCA offers improvement in the performance of LDA, MLE, and SVM classifiers which is not statistically significant. As a consequence, TD features and feature sets MAV, STD, WL, DAMV, IAV, and FS in combination with KNN and FS/MLE are recommended to obtain higher recognition accuracy rates while maintaining low processing times, whereas LogRMS and NLE in combination with LDA and MLP are suitable when time consumption is not a key requirement.

## Abbreviations

ANN: Artificial neural networks
AR: Autoregressive
Cor: Correlation coefficient
DAMV: Difference absolute mean value
DASDV: Difference absolute standard deviation value
DWT: Discrete wavelet transform
EMG: Electromyography
Er: Energy
FFT: Fast Fourier transform
FDim: Fractal dimension
FDF: Frequency domain features
FE: Frequency energy
FR: Frequency ratio
FS: Feature set
HFD: Higuchi’s fractal dimension
Hist: Histogram
HCom: Hjorth complexity parameter
HMob: Hjorth mobility parameter
IAV: Integrated absolute value
KNN: K-nearest neighborhood
Kurt: Kurtosis
LDA: Linear discriminant analysis
LogRMS: Logarithmic root mean square
MAV: Mean absolute value
MAVS: Mean absolute value slope
MaxAV: Maximum absolute value
MDF: Median frequency
MFL: Maximum fractal length
MFV: Mean firing velocity
MLE: Maximum likelihood estimation
MNF: Mean frequency
MPK: Mean of peaks
MPV: Mean of peak values
MLP: Multilayer perceptron
MM: Muscular model
MPR: Myoelectric pattern recognition
mV: Millivolt
NLE: Normalized logarithmic energy
NP: Number of peaks
PCA: Principle component analysis
Perc: Percentile
PKF: Peak frequency
RE: Relative energy
DT: Decision tree
s: Seconds
Skew: Skewness
SSC: Slope sign changes
STD: Standard deviation
STDPK: Standard deviation of peaks
SVM: Support vector machine
TDAR: Combined TD and AR
TDF: Time domain features
TFDF: Time-frequency domain features
Var: Variance
WAM: Willison amplitude
WL: Waveform length
WPT: Wavelet packet transform
ZC: Zero crossing
